# Stress induced by perceived radiological risk among imaging department staff

**DOI:** 10.1192/j.eurpsy.2025.2339

**Published:** 2025-08-26

**Authors:** A. Haddar, I. Sellami, M. A. Ghrab, A. Feki, M. Hajjaji, M. L. Masmoudi, K. Jmal Hammami

**Affiliations:** 1 Occupational Medicine, University Hospital Hedi Chaker; 2 LR/18/ES-28, University of Sfax; 3 Rheumatology, University Hospital Hedi Chaker, Sfax, Tunisia

## Abstract

**Introduction:**

Healthcare-workers in medical imaging departments face a variety of professional challenges, including radiological risk, biomechanical constraints, and heavy workload. These cumulative constraints make the staff of these departments particularly vulnerable to stress.

**Objectives:**

The aim of this study was to evaluate perceived stress among radiology technicians and evaluate its associated factors.

**Methods:**

A cross-sectional study was conducted among the staff of a medical imaging department in Sfax in April 2024 during periodic visits. The Perceived-Stress-Scale-10 (PSS-10) questionnaire was used to assess perceived stress. Radiation safety training level (TL), Radiation risk level (RL) and radiation protection level (PL) were auto-evaluated on a scale of 0 to 10.

**Results:**

Our population consisted of 32 paramedical staff, 80% of whom were radiology technicians. The median age was 37 with an interquartile range (IQR) [36; 43]. The sex ratio was 0.28. The median seniority in the job was 5.5 years IQR [4; 8]. The median TL, RL and PL were 3 IQR [2; 5], 6 IQR [5; 7] and 5 IQR [3; 6] respectively. The mean PSS-10 score was 19.3±4.9. In bivariate analysis, the PSS-10 score was inversely correlated with TL (r=-0.622; p=0.001), RL (r=-0.248; p=0.213) and correlated with PL (r=0.458; p=0.016).

**Conclusions:**

Periodic visits in occupational medicine are an opportunity to detect perceived stress in this population and to strengthen their knowledge about radiation protection in order to ensure a healthier and safer working environment.

**Disclosure of Interest:**

None Declared

